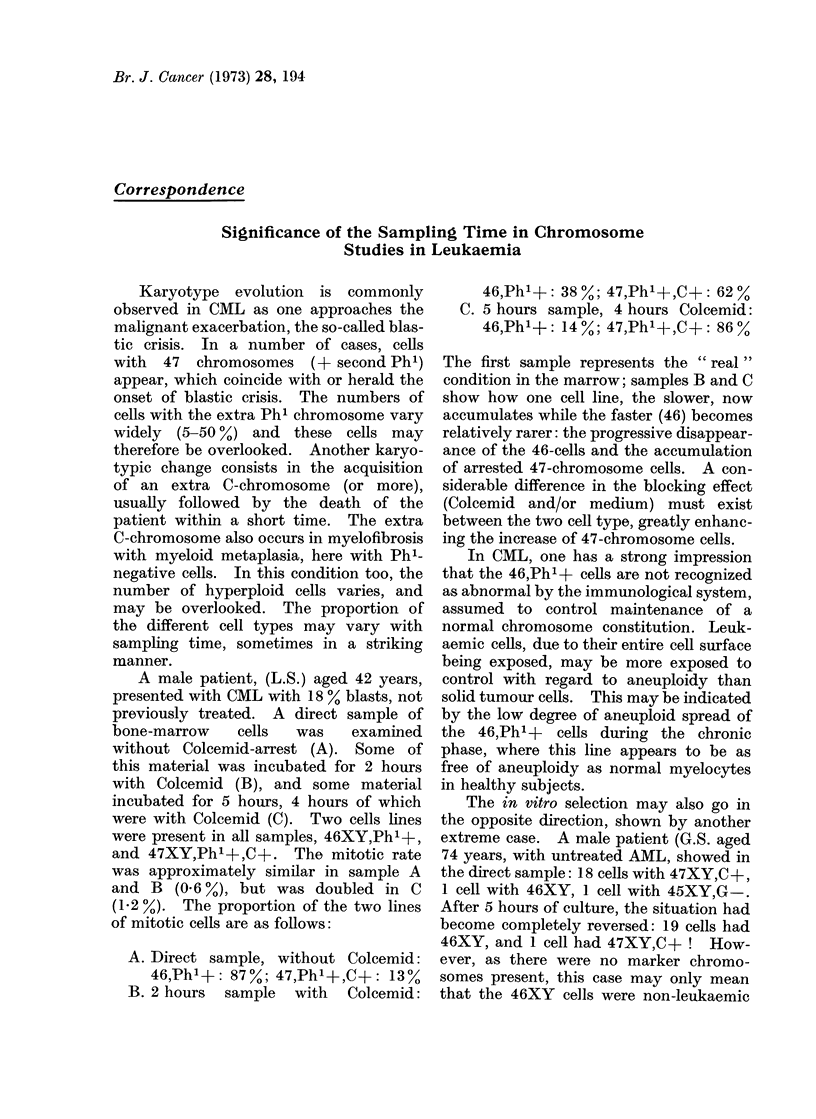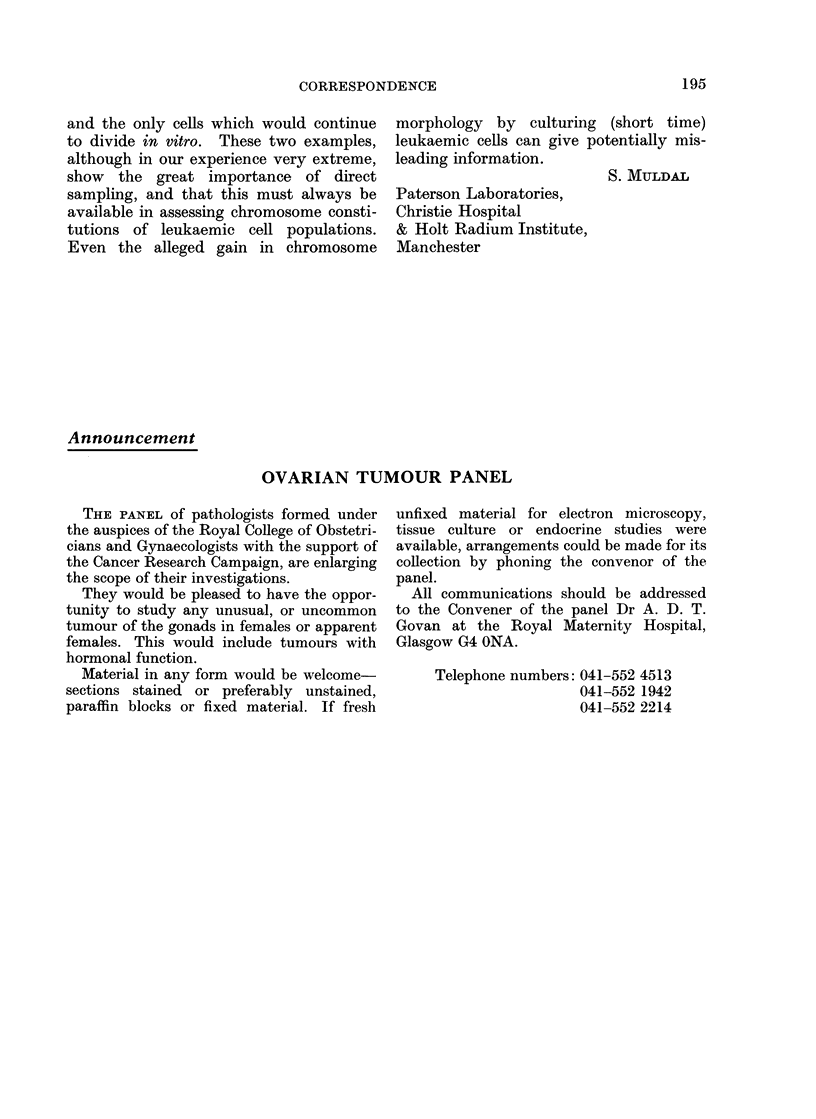# Significance of the sampling time in chromosome studies in leukaemia.

**DOI:** 10.1038/bjc.1973.137

**Published:** 1973-08

**Authors:** S. Muldal


*Br. J. Cancer* (1973) **28**, 194

### *Correspondence*

### Significance of the Sampling Time in Chromosome Studies in Leukaemia

Karyotype evolution is commonly observed in CML as one approaches the malignant exacerbation, the so-called blastic crisis. In a number of cases, cells with 47 chromosomes (+ second $Ph^1$) appear, which coincide with or herald the onset of blastic crisis. The numbers of cells with the extra $Ph^1$ chromosome vary widely (5–50%) and these cells may therefore be overlooked. Another karyotypic change consists in the acquisition of an extra C-chromosome (or more), usually followed by the death of the patient within a short time. The extra C-chromosome also occurs in myelofibrosis with myeloid metaplasia, here with $Ph^1$-negative cells. In this condition too, the number of hyperploid cells varies, and may be overlooked. The proportion of the different cell types may vary with sampling time, sometimes in a striking manner.

A male patient, (L.S.) aged 42 years, presented with CML with 18% blasts, not previously treated. A direct sample of bone-marrow cells was examined without Colcemid-arrest (A). Some of this material was incubated for 2 hours with Colcemid (B), and some material incubated for 5 hours, 4 hours of which were with Colcemid (C). Two cells lines were present in all samples, 46XY,$Ph^1$+, and 47XY,$Ph^1$+,C+. The mitotic rate was approximately similar in sample A and B (0·6%), but was doubled in C (1·2%). The proportion of the two lines of mitotic cells are as follows:

A. Direct sample, without Colcemid: 46,$Ph^1$+ : 87%; 47,$Ph^1$+,C+ : 13%

B. 2 hours sample with Colcemid: 46,$Ph^1$+ : 38%; 47,$Ph^1$+,C+ : 62%

C. 5 hours sample, 4 hours Colcemid: 46,$Ph^1$+ : 14%; 47,$Ph^1$+,C+ : 86%

The first sample represents the "real" condition in the marrow; samples B and C show how one cell line, the slower, now accumulates while the faster (46) becomes relatively rarer: the progressive disappearance of the 46-cells and the accumulation of arrested 47-chromosome cells. A considerable difference in the blocking effect (Colcemid and/or medium) must exist between the two cell type, greatly enhancing the increase of 47-chromosome cells.

In CML, one has a strong impression that the 46,$Ph^1$+ cells are not recognized as abnormal by the immunological system, assumed to control maintenance of a normal chromosome constitution. Leukaemic cells, due to their entire cell surface being exposed, may be more exposed to control with regard to aneuploidy than solid tumour cells. This may be indicated by the low degree of aneuploid spread of the 46,$Ph^1$+ cells during the chronic phase, where this line appears to be as free of aneuploidy as normal myelocytes in healthy subjects.

The *in vitro* selection may also go in the opposite direction, shown by another extreme case. A male patient (G.S. aged 74 years, with untreated AML, showed in the direct sample: 18 cells with 47XY,C+, 1 cell with 46XY, 1 cell with 45XY,G−. After 5 hours of culture, the situation had become completely reversed: 19 cells had 46XY, and 1 cell had 47XY,C+ ! However, as there were no marker chromosomes present, this case may only mean that the 46XY cells were non-leukaemic and the only cells which would continue to divide *in vitro*. These two examples, although in our experience very extreme, show the great importance of direct sampling, and that this must always be available in assessing chromosome constitutions of leukaemic cell populations. Even the alleged gain in chromosome morphology by culturing (short time) leukaemic cells can give potentially misleading information.

S. Muldal

Paterson Laboratories,
Christie Hospital
& Holt Radium Institute,
Manchester